# Structured communication methods for mental health consultations in primary care: a scoping review

**DOI:** 10.1186/s12875-023-02129-y

**Published:** 2023-09-04

**Authors:** Franziska Mosler, Katy Packer, Lauren Jerome, Victoria Bird

**Affiliations:** 1 Queen Mary University of London, London, UK; 2 University College London, London, UK

**Keywords:** Primary care, Mental health, Structured communication

## Abstract

**Background:**

Majority of people with mental health problems attend primary care for support. Interventions that structure consultations have been found effective for physical health conditions and secondary mental health care. The aim of the review is to identify what tools or interventions exist to structure communication in primary care for appointments related to mental health problems and examine existing evidence for effectiveness for mental health and quality of life outcomes.

**Methods:**

Quantitative and qualitative studies were eligible for inclusion if staff was based in a primary care setting and the intervention involved bi-directional communication with adult patients. Six databases were searched (MEDLINE, Web of Knowledge, EMBASE, PsycINFO, The Cochrane Library, CINAHL) with no time restriction. Search terms combined four concepts with key words such as “structured” and “interaction” and “mental illness” and “primary care”. Reference lists of eligible studies were searched.

**Results:**

After removing duplicates, 3578 records were found and underwent further screening. A total of 16 records were included, representing eight different interventions from five countries. The majority were delivered by primary care doctors and focused on patients experiencing psychological distress. Similarities across interventions’ service delivery were that most were created for a broad patient population, used self-report assessments at the start and actions or plans as the end point, and employed group settings and didactic methods for training staff in the intervention. Booster and follow-up trainings were not offered in any of the interventions, and supervision was only part of the process for one. The evidence for effectiveness for mental health and quality of life outcomes was mixed with three out of five RCTs finding a positive effect.

**Conclusions:**

Although the idea of structuring communication for mental health consultations has been around since the 1980s, relatively few interventions have attempted to structure the conversations within the consultation, rather than modifying pre-visit events. As the evidence-base showed feasibility and acceptability for a number of interventions, there is scope for those interventions to be developed further and tested more rigorously.

**Supplementary Information:**

The online version contains supplementary material available at 10.1186/s12875-023-02129-y.

## Background

Structured communication tools are one way that health care providers have been attempting to improve patient-centred communication to affect outcomes such as psychological symptoms, and adherence to and satisfaction with treatment [1].

Physical healthcare settings have used structured communication tools as a way of ensuring high quality care [2]. For example, in the context of palliative care, to shift conversations away from clinician-led discussions of treatment options towards care planning around patients’ goals and values [3] or to improve satisfaction with care and create realistic expectations in relatives with a critically ill family member [4].

Mental health care is predominantly delivered by primary care providers [5]. Structured agendas have previously been identified as an added benefit of a psychological intervention by GPs [6]. Research into structured communication in primary care has focused on pre-consultation tools that can be broadly categorised into “question prompt lists” that include some form of systematic lifestyle or mental health assessment and a further patient-centred, issue prioritisation step [7–10]. Results have shown feasibility and acceptability for such approaches and some promise for better identification of mental health issues but no immediate impact on patient outcomes.

While in secondary mental health care interventions that structure the care coordination meetings themselves have been found effective [11], it is less clear what research has been conducted to structure consultations around mental health concerns between patient and clinician in primary care settings. We therefore conducted a scoping review with the aim to create an overview of what interventions or tools exist in the literature, what is known about their effectiveness, and how they are described in terms of intervention content and delivery.

This review therefore aimed to answer the following questions;

I. What structured communication tools are used in primary care for mental health consultations and what are common features or components in terms of intervention content and delivery?
II. What is the evidence of effectiveness for mental health and quality of life outcomes?

## Methods

A scoping review methodology with mixed studies inclusion was conducted. The research question on evidence of effectiveness only considered quantitative outcome data.

The Joanna Briggs Institute (JBI) and Preferred Reporting Items for Systematic Reviews and Meta-analyses extension for scoping review (PRISMA-ScR) guidelines were followed [12, 13].

### Search methods

A three-step search strategy as recommended by JBI guidelines [13] was followed. In the first step an initial limited search of MEDLINE and Web of Knowledge databases was carried out to analyse text words and index terms of relevant papers. In the second step the following databases were searched with all previously identified keywords and index terms: MEDLINE, Web of Knowledge, EMBASE, PsycINFO, The Cochrane Library, CINAHL May 2021. Search terms combined four concepts with key words such as “structured” AND “interaction” AND “mental illness” AND “primary care”” (see Additional file 1). In the third step, references of eligible studies and appropriate reviews were searched for additional citations.

### Study selection

All identified citations were collated and uploaded into EndNote X8.2 [14] and duplicates removed. Titles were screened by the first author (FM) to identify possible articles for full text retrieval and a second reviewer (KP) assessed 10% of the citations independently.

Abstracts and full texts were read and chosen for inclusion by FM with 25% of papers assessed independently by second reviewer KP. Any discrepancies or disagreements were resolved by discussion and consensus, and when in doubt, the final decision was made in consultation with a third reviewer VB. Reasons for exclusion at the full text stage were recorded.

The inclusion criteria were published and unpublished full texts of empirical quantitative and qualitative studies, published in any language using the Latin alphabet. For interventions to be included, patient participants had to be aged 18 years and over and attend primary care with a mental health problem. Staff had to be located in primary care services but did not need to be registered health care professionals. Data on effectiveness was only included from peer-reviewed publications & doctoral theses.

The intervention or tool of interest had to either have the explicit aim to structure communication or follow concrete steps, e.g. decision aids, action planning, agenda setting etc. Communication had to be bi-directional between patient and staff participants, this could either be face-to-face or remote. The content of the consultation had to be mental health focused. Records were excluded if the interventions were described as self-help programmes, online chats, group approaches, or psychotherapy.

### Data extraction

Data extraction was performed primarily by FM, with second reviewer (KP) extracting one article for both extraction tables – study characteristics and service delivery.

Extraction included information such as author(s), year of publication, country, clinical setting, study design/methods, aims, patient and staff participant numbers & inclusion criteria such as mental health conditions and professional groups, intervention and control group descriptions, outcomes and measures, follow up times, primary/secondary and qualitative findings.

All quantitative outcomes were of interest; however, only mental health and quality of life measures would be looked at in terms of effectiveness for research question two.

For intervention content and delivery, data extracted included whether there was integration into electronic patient records, availability as an app, other materials provided, whether GPs deliver the intervention or other professionals, target patient population, underlying theoretical orientation, delivery in-person or remote, length of consultations, planned follow ups provided, use of a self-assessments to inform consultations, use of templates to guide conversation, planned repeated sessions, setting of actions, training, and opportunity for supervision.

If a feature from Table 2 wasn’t explicitly mentioned in the article or additional materials provided by the authors, it was rated as not present. Authors were contacted to request missing or additional data.

### Quality assessment

Separate tools for assessing the quality of quantitative and qualitative research designs were chosen. Quality assessment for quantitative designs was done using the checklist developed by the Effective Public Health Practice Project [15]. It has established validity and reliability [16] and has been judged to be suitable for reviews of effectiveness [17]. The qualitative study was assessed using the Critical Appraisal Skills Programme checklist for qualitative research [18] which has been used widely in health research evidence synthesis [19].

Any mixed method papers would also be assessed using one of the two tools as the criteria of success at integration of methods is of no concern to the scoping review. KP independently assessed one article for each checklist (14%).

### Data analysis

For research question I (RQI) and RQ II, studies were summarised and tabulated in terms of their characteristics and outcomes. Additionally, each intervention was charted according to intervention content and service delivery features.

## Results

**Fig. 1:**
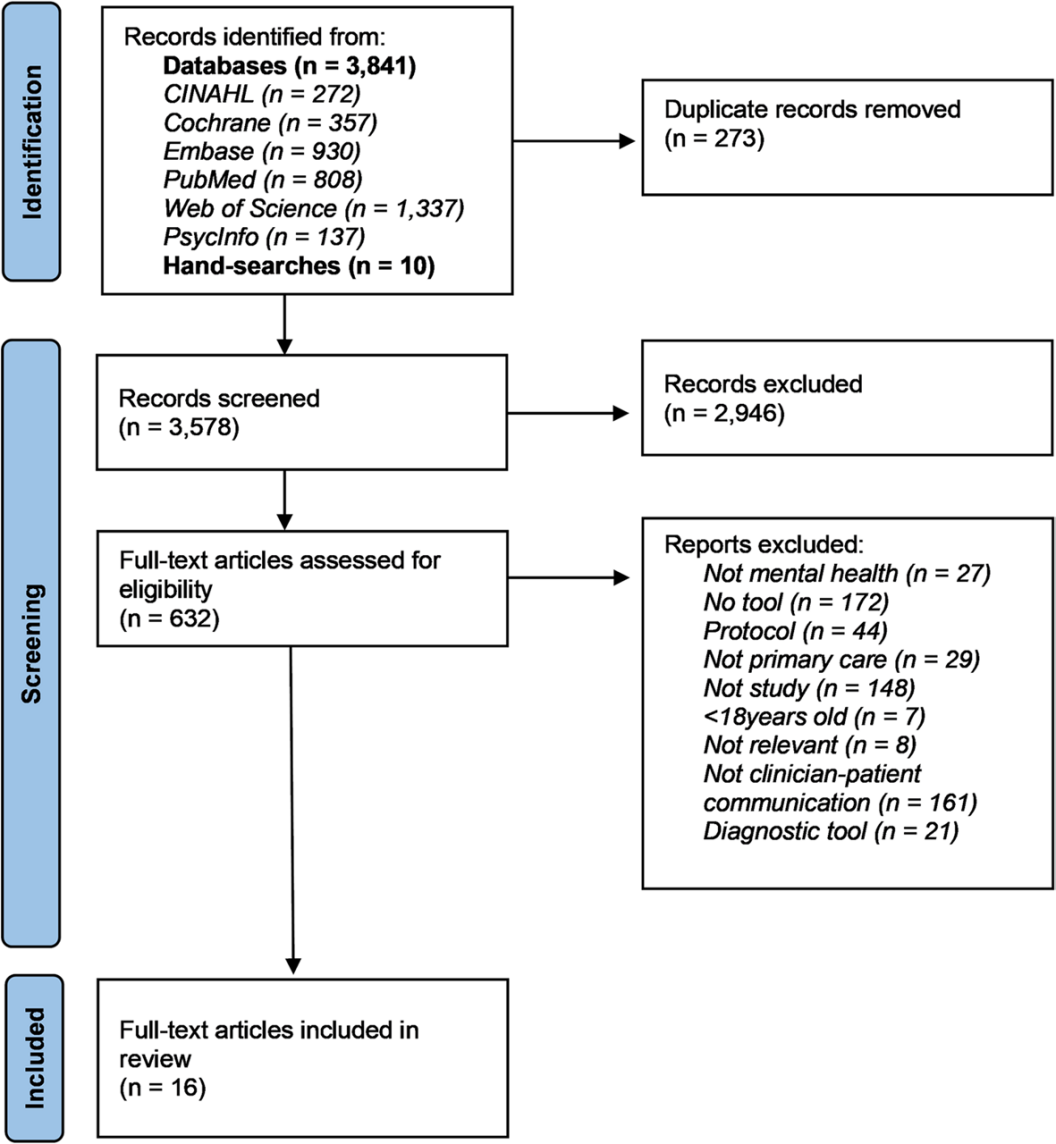
PRISMA-ScR flow diagram

The search strategy found 3,841 records through databases and ten through hand-searches. A total of 273 records were removed as duplicates, and a further 2,946 records were excluded at the title screening stage for not meeting the inclusion criteria. The full texts of the remaining 632 articles were examined and a total of 16 included. They represented 14 unique studies and eight distinct interventions. One study was reported in three articles [20–22]. The PRISMA-ScR flow diagram (Fig. 1) shows the article selection process in detail. All full-texts were available thus no authors were contacted for access, but three authors were contacted to request missing or additional data [21, 23, 24] with one responding [23].

### RQ I—What structured communication tools are used in primary care for mental health consultations and what are common features or components in terms of intervention content and delivery?

The search found eight interventions. The articles were published between 1989 and 2021, half of them within the last decade. Interventions were developed in the USA (*n* = 4), New Zealand (*n* = 1), UK (*n* = 1), Spain (*n* = 1), and Hong Kong (*n* = 1). The characteristics of the studies are summarised in Table 1 and the interventions briefly described below

- 1. *predictD Intervention*. The intervention is the extension of the predictD tool that was developed to accurately predict the occurrence of major depression at twelve months, using data entered by patients regarding twelve risk factors (e.g. sex, age, childhood physical abuse, health-related quality of life) [25].
  As part of the intervention, primary care physicians attended training workshops on depression and how the predictD intervention applies to clinical case examples. Physicians offered three sessions to patients who scored at moderate to high risk on the predictD tool during which they provided a tailored bio-psycho-social intervention. Physicians are given a seven-item list of recommendations to activate and empower patients during those consultations. Additionally, patients are offered a booklet about preventing depression.
- 2. *Feedback/Feedback + counselling*. Before attending their consultation, patients would fill in the General Health Questionnaire (GHQ) and a questionnaire about their current life stress, how well they had been coping with it and how much they felt the physician could help and what specific types of things they could do to help. The primary care physicians in the “feedback” intervention group were provided with patients’ GHQ score and an explanation of the probability of them having a mental health disorder.
  Physicians in the “counselling protocol” group were, additionally to the feedback, provided with a protocol which first listed questions to evaluate the stressful situation further and elucidate strategies patients use to cope in the past and present. Second, the protocol listed counselling interventions for the physician to choose, e.g. problem solving, restructuring patient attitudes, and effective coping strategies.
- 3. *Self-Efficacy Enhancing Interviewing Techniques (SEE IT)*. The intervention aims at teaching residents and primary care physicians interviewing techniques that would enhance patients’ self-efficacy in achieving health behaviour changes. SEE-IT consists of nine discreet components that are presented to the physicians as a process flow chart, i.e. the conversations start at “1. Solicit all of the patient’s concerns up front” and ends in “9. Negotiate when and how patient will follow-up with you on behaviour change progress”. Components 4, 5, and 7 have answer options which either skip components or move the conversation back to previous components. For example, “5. Assess confidence to take this step [towards the behaviour change goal]” can either be answered as “high” skipping ahead to component “8. Check for understanding of behaviour change plan” or “low” moving back to component “2. Negotiate behaviour change goal to focus on”.
- 4. *Problem-Solving Treatment in Primary Care (PST-PC)*. This is a brief, three session intervention targeting elderly patients with undiagnosed psychological problems. They are asked to complete the Hospital Anxiety and Depression Scale (HADS) prior to the consultation.
  Family medicine trainees have a proforma to complete HADS scores and symptoms, circle what they thought is the main psychological diagnosis, and record somatic symptoms and problems in living. Then the form would prompt them to “Ask the patient to identify their main problem” and rate it on a ten-point scale from very mild to extremely severe. Following this there was a six-step “solution plan” starting with “ask patient to think of possible solutions” and ending with “patient is to work on first step of their preferred solution and report progress to you”. The forms for the two follow-up sessions started by asking patients to rate the severity of their main problem again and answer two additional questions on what they have done to solve their problems since the last session and whether it was effective, before returning to the previous six-step plan. The authors reported that the sessions had three core tasks to achieve: “establishment of a positive therapeutic relationship, developing a shared understanding of the problem, and promoting change in behaviour, thoughts, and emotions” (p.971 [26]).
- 5. *Ultra-Brief Intervention (UBI)*. The authors describe this intervention as “guided, cognitive behavioural therapy-based self-management, with a focus on problem solving and behaviour change” (p.232 [27]). Patients were identified as having sub-threshold psychological distress by completing the Kessler-10 questionnaire. They were offered three sessions which were structured by a series of questions asked by the clinician in order to a) clarify the problem b) identify coping strategies c) create written plan of action d) and build motivation to carry out actions. Patients were given actions plans printed out as prescriptions and after the in-person sessions there would be one follow-up phone call or email.
- 6. *Shared Decision Making*. This intervention introduced shared decision making into medication therapy management consultations between pharmacists and patients prescribed at least three medications. A conversation template with nine distinct steps was integrated into the electronic patient record. The template prompts started with eliciting patient concerns, preferences, values and goals, then move on to pharmacist assessment, patient and pharmacist identified solutions, a decision, communication of the plan, and lastly a follow-up. The initial step of patient reported concerns had pre-set options in a drop-down menu such as “medication cost” or “side effect”, as did the eighth step of “communication of plan”, e.g. “patient will take recommendation to provider” or “no action”.
- 7. *Reattribution*. This was a structured cognitive approach for patients with diagnosed mental health disorders who presented with somatic symptoms in primary care during routine appointments. The two studies included in the review used a three- step model of reattribution which also contains several suggested sub-components. First step “feeling understood”: GPs would gain an understanding of patient’s complaint by taking a comprehensive history, responding to mood cues, exploring health beliefs and carrying out a physical examination. Second step “broaden the agenda”: GP would reframe physical complaint by summarising physical findings, acknowledge reality of complaint (e.g. pain), and reminding patient of other symptoms and life events. Lastly “Making the link”: GP would make the link between patient distress and physical complaint by explaining anxiety and depression, demonstrating the link practically, in terms of life events, or making explicit what is happening in the here and now or projecting onto a family member. A decade later, further refinement of the intervention led to the addition of a fourth step called “negotiating treatment” into the model [28].
- 8. *Peer Coaching*. Veteran peer coaches would have up to four phone calls with veterans who screened positive for at least one mental health disorder but were not currently in treatment. Coaches would follow a motivational interviewing structure, after initial sharing of results of the mental health questionnaires. The target behaviour change was initiation of mental health treatment or, if that was achieved, treatment retention. Additional coaching language phrases around personal values and goals were also included.

**Table 1:** Characteristics of included studies

**Author(s), year of publication/country**	**Design/ methods**	**Main objective**	**Patient mental health criteria (n intervention vs control)**	**Staff profession (n)**	**Outcomes**	**Main findings**	**Quality (EPHPP/ CASP)**
Bellon et al. 2016 [21]; Fernandez et al. 2018 [22]; Moreno-Peral et al. 2021 [20]/ Spain	RCT/ Quantitative	Can intervention delivered in primary care settings prevent depression	Moderate/high risk of depression (1663 vs 1663)	Primary care physicians (140)	Incidence of major depression; incidence of anxiety; cost-effectiveness	No difference in incidence of depression; lower incidence of anxiety in intervention group; very likely cost-effective	Strong
Brody et al. 1990 [29]/ USA	RCT/ Quantitative	Evaluate the impact of two types of interventions on the primary care physician's management of patients with mental health problems	Mental health problems, ≥ 3 on GHQ (29 vs 24 vs 50)^a^	Internal medicine resident (60)	Patients: discussion of stress; compare pre-visit to post-visit attitudes about their stress; satisfaction with care; residents: care provided	More valuable stress counselling and more satisfied with their physician compared with control group; greater perceived reductions in the amount of stress and greater increases in their sense of control over stress; no difference in care provided by residents	Weak
Collings et al. 2012 [30]/ New Zealand	Cohort/ Quantitative	Acceptability of ultra brief intervention	Mental health problems, > 35 on Kessler-10 (19)	GPs & nurses (6)	Patient & clinician satisfaction; psychological distress	High levels of acceptability; improvement in distress	Moderate
Gask et al. 1989 [31]/ UK	Cohort/ Quantitative	Effectiveness of training in reattribution skills	Standardised patients (3)	GP trainees (22)	Increase in use of three steps of reattribution	Improvement in one step (“Making the Link”)	Moderate
Jerant et al. 2009 [32]/ USA	RCT/ Quantitative	Effectiveness of intervention for training residents in SEE IT	Standardised patients (4)	Family medicine, internal medicine (64)	Use of SEE IT by residents; socio-demographic; training acceptability	Greater use of SEE ITs, training acceptable	Weak
Jerant et al. 2016a [33]/ USA	RCT/ Quantitative	Effectiveness of intervention for training physicians in SEE IT	Standardised patients (6)	Family physicians, general internists (28 intervention vs 24 control)	Use of SEE IT; response to training	Greater use of SEE ITs; higher training value; similar low hassle	Weak
Jerant et al. 2016b [34]/ USA	Case control/ Quantitative	Does exposure to SEE IT enhance patient self-efficacy and health behaviour change mediators	Mental health problems, ≥ 10 on PHQ-9 (131)	As above	Self-care self-efficacy; readiness for self-care of health conditions; health locus of control; socio-demographic; health indicator variables, depression symptoms	More favourable post-visit scores on a composite measure of five psychological HBCMs—driven by increased stage of readiness for self-care and reduced Chance health locus of control	Moderate
Lam et al. 2010/ Hongkong [26]	RCT/ Quantitative	Effectiveness in improving quality of life and reducing consultation rates	Mental health problems, positive screen on HADS (149 vs 183 vs 150)^a^	Family medicine trainees	HRQoL; mental health; consultation rate; trainees’ competences	Same improvement in HRQoL; same decrease in mental health severity; trainees used core techniques 90% of sessions	Moderate
Mathieson et al. 2013 [35]/ New Zealand	Collaborative/ Qualitative	Develop brief intervention	Sub-threshold depression or anxiety (14)	Doctors & nurses (15)	n/a	CBT-based guided self-management approach; three sessions over 5 weeks	7/9 criteria met
Mathieson et al. 2012 [27]/ New Zealand	Cohort/ Mixed	Acceptability of ultra brief intervention for Maori population	Mental health problems, > 35 on Kessler-10 (22)	GPs & nurses (23)	Adaptations made; Patient & clinician satisfaction; psychological distress	Addition of Maori language and concepts to intervention; 56% completed intervention; positive feedback; improvement in distress	Weak
Mathieson et al. 2019 [23]/ New Zealand	RCT/ Quantitative	Effectiveness of ultra brief Intervention In improving mental health and functioning	Mental health problems, > 35 on Kessler-10 (85 vs 75)	GPs (62 vs 50)	Psychological distress; anxiety/depression: work, social and relationship functioning	No difference in psychological distress & secondary outcomes; unable to achieve full recruitment to sample size	Moderate
Montag Schafer et al. 2016 [24]/ USA	Cohort/ Mixed	Effectiveness, feasibility, acceptability of intervention	Diagnosed mental health disorder (20)	Pharmacists (8)	Patient & pharmacist satisfaction; number of drug therapy problems	Positive feedback from patients, mixed from pharmacists; average 2 DTP identified	Weak
Morriss et al. 1998 [36]/ UK	Static group comparison/ Quantitative	Cost-effectiveness of training GPs in reattribution	Somatised mental health disorder & GHQ-12 > 3 (112 vs 103)	GPs (8)	Self-rated psychiatric symptoms; direct health costs	No difference in psychiatric cases; total costs reduced by 15%	Moderate
Seal et al. 2021 [37]/ USA	RCT/ Mixed	Effectiveness in improving mental health treatment engagement among veterans	Screened positive for ≥ 1 mental health problem & not engaged in treatment (137 vs 135)	Veteran peer coaches (2)	Initiation of mental health treatment and retention; other care; mental health symptoms & QoL; patient experiences; fidelity	No difference in treatment initiation & retention; more intervention participants engaged in other activities; fewer MH symptoms, better QoL; fidelity was 3/5	Moderate

*RCT* randomised controlled trial

^a^Two intervention groups; *GHQ* General Health Questionnaire, *SEE IT* Self-efficacy enhancing interviewing techniques, *HADS* Hospital Anxiety and Depression Scale

The majority of interventions was not integrated into electronic patient records (5/8). In terms of additional materials, two interventions provided patient booklets [21, 38] focusing on psychoeducation and self-help strategies.

Six interventions were specifically developed for the use by primary care physicians, whereas Montag Schafer et al. [24] focused on pharmacists and Seal et al. [37] on veteran peer coaches.

Half of the interventions worked on the basis of diagnosed mental health conditions, whereas the other half focused on psychological distress. The only intervention restricted to a particular mental health diagnosis was the predictD intervention by Bellon and colleagues [21] that targeted risk of major depression instead of existing cases. Two interventions were designed for specific sections of the population – veterans [37] and people aged 60 years or over [26].

The terms “motivational interviewing” and “problem-solving” appear most frequently to describe the underlying therapeutic approach of the structured interventions. The bio-psycho-social model and cognitive behavioural therapy each appear twice.

All but one intervention had been developed to be used during in-person meetings. The feedback/ + counselling intervention [29] took the least amount of consultation time with less than 5 min reported. Shared decision making in medication therapy management [24] had the longest consultation time lasting from 30 up to 60 min. Half of the interventions had a repeated sessions design, mostly three [21, 23, 26] but peer coaching allowing up to four [37].

Six interventions made use of a self-report assessment as the starting point for the consultation. Out of those six, four made use of a mental health screener. The ultra-brief intervention (UBI) had the Kessler-10 as starting point [23, 27, 30, 35] and the peer coaching interventions screened for five target mental health disorders ahead of the appointment [37]. The feedback/ + counselling intervention used the GHQ as well as a patient assessment of their level of stress and any help they require with it [29]. The problem-solving treatment in primary care (PST-PC) involved the HADS as well as patients being asked to identify their main problem and rate its severity on a 10-point scale. Beyond those, the predictD intervention asked participants to complete a whole range of questions about their past and current life as well as a health-related quality of life measure (SF-12) [25]. Whereas the shared decision-making tool prompts clinicians to ask for the presence of frequently reported medication concerns [24].

The format of completing a patient self-reported assessment as part of the intervention consultation with the clinician was in the minority [24, 26]. Actions, goals, or plans were agreed or made as part of five interventions [23, 24, 26, 32, 37] and seemed implied but not explicitly mentioned for two [21, 29].

#### Training, supervision & fidelity

All interventions provided in-person training but only five gave details on the length of sessions. The SEE-IT intervention [32, 34] had the shortest reported time with three 20 min sessions, and the predictD intervention the longest with 15 h of training. Training was carried out by a range of different professional groups with psychologists being the most prevalent [23, 26, 29, 37].

Most authors described didactic teaching methods such as presentations, manuals, and reading materials. Three interventions additionally employed application-oriented experiences through simulated patients, role plays, and case discussions [21, 23, 33]. Group settings were most frequently used to train, with only two studies describing one-to-one instructions [29, 32, 33].

None of the interventions offered booster/top-up training.

Peer coaching [37] was the only intervention offering supervision.

Five intervention studies had no fidelity or adherence checks as part of their study design. For the two interventions which did check for fidelity, the peer coaches showed “fair fidelity” to MI techniques [37] and GPs achieved the objectives of problem-solving treatment in more than 83% of sessions [26]. The SEE IT [32] and reattribution [31] interventions had separate studies to specifically investigate whether clinicians applied the skills they were previously trained in.

The eight interventions are described regarding the presence of features related to service delivery in Table 2.

**Table 2:** Content and service delivery by intervention

	**Bellon et al**	**Brody et al**	**Jerant et al**	**Lam et al**	**Mathieson et al**	**Morriss et al**	**Montag Schafer et al**	**Seal et al**
**Intervention**	**predictD** **Intervention**	**Feedback/ + Counselling**	**SEE-IT**	**PST-PC**	**UBI**	**Reattribution**	**Shared Decision Making**	**Peer Coaching**
**Integrated into EPR**	**?**	**x**	**x**	**x**	✓	**x**	✓	**x**
**Other materials**	Patient booklet	**x**	**x**	**x**	Printed out action plans; three booklets	**x**	**?**	**x**
**GPs**	✓	✓	✓	✓	✓	✓	**x**	**x**
**Other profession**	**x**	**x**	**x**	**x**	**x**	**x**	Pharmacists	Veteran peers
**Target population**	Moderate to high risk for depression	Mental health problems	Not specified	Elderly with unrecognised psychological problems	Psychological distress	Somatised mental disorder	≥ 1 mental health disorder; ≥ 3 medications	Veterans with ≥ 1 mental health conditions
**Orientation**	Bio-psycho-family-social	Feedback/ Problem-solving, restructuring, coping strategies	Self-efficacy, MI, behavioural theories	Problem solving	Problem solving, MI, CBT	Bio-psycho-social	Shared decision making	MI, Coaching
**In-person/remote**	In-person	In-person	In-person	In-person	In-person	In-person	In-person	Remote
**Consultation time**	10 min	≤ 5 min	20 min	20-45 min	30 min, 2 × 15 min	-	30-60 min	20-30 min
**Follow-up by clinician**	**x**	**x**	**x**	**x**	Phone call/email	**x**	✓	**x**
**Self-report assessment**	✓	✓	**x**	✓	✓	**x**	✓	✓
**Timing of self-assessment**	Prior to consultation	Prior to consultation	**x**	Part of consultation	Prior to consultation	**x**	Part of consultation	Prior to consultation
**Conversation template**	✓	✓	✓	✓	✓	✓	✓	✓
**Repeated sessions**	✓	**x**	**x**	✓	✓	**x**	**x**	✓
**Actions/Goals/Plan**	**?**	**?**	✓	✓	✓	**x**	✓	✓
**Training**	10-15 h	Brief one-to-one	20 min × 3	9 h	2 h	8 h	Yes – no time given	Yes – no time given
**Training approach**	Role-play; video comments; discussion	Overview of protocol; reading material	Standardised Patient visits; 7 min consultation; 13 min scripted teaching; visual aid	Three workshops; reading materials	Presentation; video demonstration; role-play practice; discussion; manual	Instructional video; detailed teaching; role play; video feedback in small group; written information	Overview of SDM theory & template	Not described
**Trainer**	**?**	MD & clinical psychologist	Physician assistant	Clinical psychologist	Psychologist & PCP	**?**	Pharmacists & nurse practitioner	Psychologist
**Supervision/ Booster Training**	**x**	**x**	**x**	**x**	**x**	**x**	**x**	✓

*MI* Motivational Interviewing, *CBT* Cognitive Behavioural Therapy, *SMI* Serious mental illnesses

### RQ II. What is the evidence of effectiveness for mental health and quality of life outcomes?

The majority of studies used quantitative methods (*n* = 10), one was qualitative only, and three mixed methods. In terms of study design, seven of the 14 studies were randomised controlled trials, a further six quasi-experimental studies, and one collaborative qualitative research. Five studies focused on patient outcomes, three on clinician outcomes, five reported on both perspectives, and two presented cost-effectiveness analyses.

Overall, five studies had no relevant mental health or quality of life outcome measures: three of them investigated methods to teach their respective structured communication tools, in this case SEE-IT and reattribution, and Montag Schafer [24] measured satisfaction with the new shared decision-making template and the number of drug related issues identified. Mathieson and others’ [35] qualitative study focused on reporting the collaborative process of developing a new intervention between clinicians, patients, and researchers.

Four studies had mental health and or quality of life outcomes but were not randomised controlled trials. The initial UBI [30], as well as the version adapted to Māori populations [27], reduced psychological distress in patients at weeks 2, 6, and 12 post-treatment (with the reduction at 12 weeks not being statistically significant in the adapted version). Jerant and others [34] found that patients, seen after physicians had received training in the SEE-IT intervention, had improved summary “health behaviour change mediator” scores compared to a control group (+ 0.42, 95% CI 0.07–0.77; *p* = 0.021). Finally, Morris et al. [36] found that training GPs in reattribution skills led to fewer of the after-training patient cohort scoring as psychiatric cases compared to the before-training (55% vs 68%), though this also did not reach statistical significance.

Out of the randomised controlled trials, five reported mental health outcomes and two reported on quality of life. There were two validated QoL measures (WHOQOL-BREF, SF-36) and nine mental health measures (CIDI; GHQ; PCL-5, PHQ-9, K10, HADS, WHO-ASSIST, Severity Measure for Panic Disorder; Severity Measure of Generalized Anxiety Disorder). One study used a non-validated mental health measure to assess “stress” [29].

Table 3 presents the findings for mental health and quality of life measures in the RCTs. Three RCTs found no significant differences between groups [21, 23, 26]. Brody and others reported improvements in patients’ attitude about stress after the feedback/ + counselling sessions [29]. Seal et al. presented significant improvements in PTSD and depression symptoms, cannabis use, as well as individual quality of life components for patients receiving peer coaching [37]. Secondary analysis of predictD RCT data showed significant reduction in the incidence of anxiety for the intervention group at 18 months [20].

**Table 3:** Mental health and QoL outcomes by RCT

**Intervention**	**Outcome (Measure)**	**Time points of outcome measurement**	**Intervention**	**n**	**Control**	**n**	**Difference** *(adjusted when available)*	**Stat. Sign**
**predictD Intervention** *(Bellon *et al. *2016* [21]*; Moreno-Peral *et al. *2021* [20]	New cases of depression (%; 95%CI)	18m	7.39 (5.85 to 8.95)	1663	9.40 (7.89 to 10.92)	1663	-2.01 (-4.18 to 0.16)	0.070
	New cases of anxiety (%; 95%CI)		10.4 (8.7 to 12.1)	1484	13.1 (11.4 to 14.8)	1514	–2.7 (–5.1 to 0.3)	0.029
**Feedback/ + Counselling** *(Brody *et al. *1990)* [29]	*Changes in patient attitude about stress (mean, SE)*							
	Amount of stress	Post consultation	3.8 (0.1)^a^ / 3.6 (0.2)^b^	29^a^ /24^b^	3.2 (0.1)	50		0.003
	Control over stress		3.7(0.1)^a^/ 3.6 (0.2)^b^		3.1 (0.1)			0.01
	Seriousness of stress		3.5 (0.1)^a^ / 3.4 (0.2)^b^		3.2 (0.1)			ns
**Problem-solving – Primary Care** *(Lam *et al. *2010) *[26]	Change in anxiety (95%CI)	12m (6wks; 3m; 6m)	-1.17 (-1.84 to -0.51)	149	-1.58 (-2.09 to -1.07)	150	0.41 (-0.14 to 0.96)	0.146
	Change in depression (95%CI)		1.13 (0.39 to 1.88)		1.4 (0.67 to 2.13)		0.01 (-0.71 to 0.74)	0.972
	*Change in quality of life (95%CI)*							
	Physical functioning	12m	-2.32 (-4.84,0.21)		-1.9 (-4.31,0.52)		-1.52 (-4.08, 1.03)	0.243
	Role Physical		2.35 (-4.56,9.26)		6.17 (-1.15,13.48)		-1.36 (-7.90, 5.19)	0.685
	Bodily Pain		-1.11 (-5.7,3.47)		7.37 (2.9,11.83)		-5.21 (-9.43, 0.99)	0.016
	General Health		2.46 (-1.46,6.38)		2.35 (-1.47,6.18)		-1.90 (-5.87, 2.07)	0.348
	Vitality		-2.18 (-6.05,1.69)		-0.9 (-4.45,2.65)		-2.00 (-5.72, 1.71)	0.291
	Social Functioning		-1.76 (-6.62,3.09)		2.67 (-1.76,7.09)		-4.21 (-8.26, -0.51)	0.043
	Role Emotional		3.13 (-4.88,11.14)		10.89 (3.97,17.81)		-9.95 (-17.5, -2.39)	0.010
	Mental Health		0.86 (-2.31,4.03)		-0.4 (-3.31,2.51)		-0.60 (-3.68, 2.47)	0.701
	Physical Component		-0.54 (2.18,1.09)		0.8 (-0.96,2.56)		-1.48 (-3.25, 0.30)	0.103
	Mental Component		0.74 (-1.26 to 2.74)		1.07 (-0.7 to 2.84)		-0.51 (-2.36 to 1.35)	0.592
**Ultra-Brief Intervention** *(Mathieson *et al. *2019)* [23]	Improvement in psychological distress (mean, 95%CI)	6m (8wks; 3m)	5.9 (4.0 to 7.8)	70	7.6 (5.5 to 9.6)	69	1.68 (− 1.18 to 4.55)	0.255
	Improvement anxiety& depression (mean, 95%CI)		5.2 (3.5 to 6.9)		7.0 (5.3 to 8.7)		1.85 (− 0.62 to 4.31)	0.149
**Veteran Peer Coaches** *(Seal *et al. *2021)* [37]	*Mental health symptoms (mean, SD)*							
	PTSD	16wks	25.1 (18.4)	137^c^	29.7 (16.7)	135^c^		0.03
	Depression		9.4 (6.2)		11.1 (6.5)			0.01
	Anxiety		1.2 (0.9)		1.3 (0.8)			0.19
	Panic disorder		0.6(0.9)		0.7 (0.9)			0.21
	*Alcohol and illicit substance use (mean, SD)*							
	Tobacco	16wks	8.8 (9.5)		9.2 (10.0)			0.73
	Alcohol		7.1 (7.7)		7.7 (8.6)			0.46
	Cannabis […]		3.1 (4.8)		4.6 (6.7)			0.01
	*Quality of life (mean, SD)*							
	Physical health	16wks	12.6 (3.7)		12.0 (3.1)			0.06
	Psychological health		13.4 (2.8)		12.7 (2.5)			0.004
	Social relationships		13.3 (3.9)		12.1 (3.8)			0.003
	Environment		14.4 (2.5)		13.6 (2.6)			0.004

*SE* standard error, *SD* standard deviation

^a^Feedback group

^b^Feedback & counselling protocol group

^c^Not specified amount of missing data; m – months; wks—weeks

Both Lam et al. [26] and Seal et al. [37] report on sub-components of quality-of-life scales as well as anxiety & depression scores. Mathieson et al. [23] provided additional anxiety & depression data. The former two included patients with an identified mental health problem whereas the latter included mild-to-moderate levels of psychological distress. Lam et al. [26] had the longest follow up period at 52 weeks, whereas Mathieson et al. [38] reported at 26 weeks and Seal et al. [37] at 16 weeks.

### Quality assessment

Across quantitative studies, Bellon et al. [21] was the only one to achieve a global rating of “strong”, seven were rated of “moderate” quality, and five studies as “weak”. The criteria that achieved the most “strong” ratings was “withdrawals and drop-outs” (9 studies); i.e. withdrawals and drop-outs were described in numbers and reasons by group and follow-up rates were 80% or higher. The most “weak” ratings (5 studies) were in the “data collection methods” criteria which requires tools to be valid and reliable.

The CASP checklist was used to assess the sole qualitative study in the review [35]. Out of the nine yes/no questions, seven quality criteria were rated as met. The two assessors agreed that neither consideration of ethical issues nor rigorous data analysis were sufficiently evidenced. Individual EPHPP and CASP scores of all studies are presented in Additional file 2.

## Discussion

### Summary

The purpose of this review was to identify which structured communication tools existed for the use of mental health consultations in primary care and establish what was known about their intervention features and effectiveness. Eight interventions were identified, with the majority delivered by primary care doctors and focused on patients experiencing psychological distress.

In terms of research methods, one of the 14 studies was qualitative, three used mixed-method designs, and the rest were quantitative exploratory studies or randomised controlled trials. The evidence for structured communication in this context was mixed with three out of five RCTs finding a positive effect. Quality of studies was mostly moderate for the quantitative designs and the singular qualitative study met seven of nine criteria. Quality of life data was available in two RCTs—one study found no differences in quality of life and the other reported significant improvement for unadjusted scores on two subscales.

Similarities across interventions’ service delivery were that most were created for a broad patient population, used self-report assessments at the start and actions or plans as the end point, and employed group settings and didactic methods for training staff in the intervention. Booster and follow-up trainings were not offered in any of the interventions, and supervision was only part of the process for one.

### Comparison to literature

#### Features of interventions

As the review showed, a varied amount of features wase combined to create the individual interventions, many of them using psychological and behavioural theories as to propose mechanisms of actions. In the case of the ultra-brief intervention the intervention was co-created between clinicians, researchers, and patient partners to maximise feasibility, acceptability, and effectiveness from the outset [35]. Interestingly, both a systematic review of interventions to alter the interaction between patients and practitioners in physical health conditions [39] and a more recent scoping review of communication strategies for providing medical information [40] found that explicit theoretical underpinnings were rare.

Restrictions on time is one of the areas of debate when addressing mental health problems in primary care consultations. Longer consultations as needed for shared decision making [24] and multiple session designs such as the Problem-solving intervention [26] are a barrier to the acceptability and likelihood of implementation into routine practice. However, there is some evidence that consultations involving psychological problems in standard care have already increased durations [41]. Other arguments for considering those interventions despite higher time investment comes from research showing that not addressing psychological concerns leads to higher health care utilisation [42], missing opportunities to address emotional concerns comes with longer visits [43], and issues with moving care onto more specialised mental health care providers [44].

In this review, six out of the eight interventions were designed to be delivered by primary care physicians. This reflects the current situation that GPs are the most frequently used providers of mental healthcare [45] but could also be an artefact of the search strategy or the result of the time lag for research to catch up with the diversified workforce in primary care. Within primary care there is a tension between the care recommended by clinical guidelines and reality of prescription of psychiatric medication or no care at all [46, 47]. Qualitative data from patients and GPs has also pointed to that when it comes to emotional concerns, the GP-patient relationship was therapeutic in itself [45]- a phenomenon structured communication interventions are trying to formalise and build on to improve outcomes.

A different angle to the limited resource issue is the up skilling of other professionals such as done with pharmacists for the shared decision-making intervention [24] and drawing in non-professionals for care provisions such as peer workers such as in the veteran coaching [37]. In terms of the former, Health Education England recently published a review of innovative roles within mental health pharmacy [48], presenting many successful NHS pilot programmes of pharmacists taking more extensive roles in mental health care. Just as the intervention developed by Montag Schafer & colleagues had done, they highlight “shared decision-making skills to support meaningful conversations with patients and carers” as one of the gaps in the current education of pharmacists (p. 16).

#### Training & supervision

The heterogeneity in how primary care staff was trained across interventions in this review was reflected in a systematic review on training methods to impart skills relevant to psychological practice [49]. Garzonis and others identified 24 studies and categorised methods into individual, group, and web-based approaches. As was the case in our interventions, GPs were predominantly trained in group settings with interactive components such as discussions and role plays. Length of training was relatively longer in their studies at 1h to 4 days which is most likely due to a difference in inclusion criteria for type of intervention.

It was not the aim of our review to come to any conclusions regarding how well clinicians implemented the intervention they were trained on and what impact that had on outcomes. However, only two interventions used fidelity or adherence checks [26, 37] and this data would have been potentially useful to clarify the mixed results for outcomes, as Mathieson and colleagues commented on themselves regarding the lack of effect of the UBI [23].

A notable difference in the primary care setting was the lack of follow up training and supervision as would be common for psychological interventions [50]. One potential reason could be their conceptualisation closer to a “tool” or “protocol” rather than psychological therapy. Losing those components would also potentially improve their acceptability by limiting the time commitment necessary.

#### Effectiveness

A systematic review in 2004 had concluded that the way practitioners and patients interact in consultations could be altered and that interventions whether aimed at patients, practitioner, or both, could affect health outcomes [39]—Griffin and colleagues arrived at this conclusion employing a wider definition of interventions and settings compared to this scoping review. They found 35 randomised controlled trials, of which 23 were based in primary care and the reported patient populations, intervention components and outcomes are similarly heterogeneous to the ones in this review.

There was a range of issues assessing the effectiveness of the structured interventions we included: a lack of specified primary outcomes and time points, lack of validated outcome measures used, and inconsistent use of fidelity/adherence measures. Choosing the right concept to measure, e.g. focusing on quality of life rather than psychiatric symptom reduction should also be a consideration. Beyond research design choices, Mathieson and others [23] described restrictions of local mental health care which led to their RCT not recruiting to the necessary sample size, a common issue for research of complex interventions in primary care [51, 52].

### Limitation

This scoping review was not registered, however methodologists are suggesting the registration of scoping review protocols in public databases to improve transparency and unbiased reporting [53].

The search strategy was limited to only English-language based databases, despite all Latin-based languages being included, which could have missed relevant publications. Grey literature sources were planned to be searched but not included in the end due to time restrictions.

The inclusion of intervention was on the basis of patient participants having a mental health diagnosis. This was a proxy for the assumption that the discussions in the consultations were around mental health. However, it excluded papers and interventions that focused on patient populations defined as “frequent attenders” or those with “medically unexplained symptoms” which were likely to also address the question above.

Features of intervention content and delivery were not based on any formal framework but common information available across articles in order to facilitate the reporting of studies [40]. Therefore, additional data relevant to clinicians and services might not have been captured.

### Future research

Based on the limited number of studies identified, there is scope for the structured communications interventions to be developed further and tested more rigorously*.* As a number of studies investigated feasibility, acceptability, or required the investigators to reflect on the lack of effect of their interventions, there have been a host of suggestions for changes regarding target populations, intervention delivery and target outcomes, all worth further research.

Although this was a mixed studies review, RQ I did not focus on primary data collected for evaluation of interventions, but rather the descriptions of the structured communication interventions and training approaches as well as the context in which they were tested. RQ II was answered using only studies with quantitative data to establish evidence of effectiveness. This led to quantitative and qualitative data not being explicitly synthesised, something which would be desirable in a review once the evidence base has increased. Meta-analyses around changes in anxiety and depression as well as quality of life scores with the currently available data would be possible but potentially premature.

## Conclusions

Although the idea of structuring communication for mental health consultations has been around since the 1980s, relatively few interventions have attempted to structure the conversations within the consultation, rather than modifying pre-visit events. The evidence-base showed feasibility and acceptability for a number of interventions, but patient outcomes were mixed if not mostly without any differences. Some of those results were explained by the authors in terms of challenges of research and implementation in primary care but there is also the possibility of ineffective training methods being used and therefore clinicians not applying the new skills as intended.

### Supplementary Information

**Additional file 1. **Keywords and index terms by database.

**Additional file 2. **Quality assessment by study.

## Data Availability

The data that support the findings of this study are available on request from the corresponding author FM.

## References

[1] Cooper LA, Ghods Dinoso BK, Ford DE, Roter DL, Primm AB, Larson SM (2013). Comparative Effectiveness of Standard versus Patient-Centered Collaborative Care Interventions for Depression among African Americans in Primary Care Settings: The BRIDGE Study. Health Serv Res..

[2] Bernacki R, Hutchings M, Vick J, Smith G, Paladino J, Lipsitz S (2015). Development of the Serious Illness Care Program: a randomised controlled trial of a palliative care communication intervention. BMJ Open..

[3] Georgia AA, Swiderski D, Chuang EH, Flattau A, Stark A (2020). This is hard work: Using a template for goals of care conversations at community health centres. J Gen Intern Med..

[4] Sviri S, Geva D, vanHeerden PV, Romain M, Rawhi H, Abutbul A (2019). Implementation of a structured communication tool improves family satisfaction and expectations in the intensive care unit. J Crit Care..

[5] Parker D, Byng R, Dickens C, McCabe R (2020). Patients’ Experiences of Seeking Help for Psychological Distress in Primary Care: Doctor as Drug, Detective and Collaborator..

[6] Wilhelmsen M, Høifødt RS, Kolstrup N, Waterloo K, Eisemann M, Chenhall R (2014). Norwegian general practitioners’perspectives on implementation of a guided web-based cognitive behavioral therapy for depression: A qualitative study. J Med Internet Res..

[7] Shah K, Corter A, Bird A, Goodyear-Smith F (2019). A primary care programme to improve identification and stepped-care support of Asians with mental health and lifestyle issues. J Prim Health Care..

[8] Goodyear-Smith F, Warren J, Elley CR (2013). The eCHAT Program to Facilitate Healthy Changes in New Zealand Primary Care. The Journal of the American Board of Family Medicine..

[9] Wittink MN, Walsh P, Yilmaz S, Mendoza M, Street RL, Chapman BP (2018). Patient priorities and the doorknob phenomenon in primary care: Can technology improve disclosure of patient stressors?. Patient Educ Couns.

[10] Hamann J, Maris N, Iosifidou P, Mendel R, Cohen R, Wolf P (2014). Effects of a question prompt sheet on active patient behaviour: A randomized controlled trial with depressed outpatients. Int J Soc Psychiatry.

[11] Priebe S, Kelley L, Omer S, Golden E, Walsh S, Khanom H (2015). The Effectiveness of a Patient-Centred Assessment with a Solution-Focused Approach (DIALOG+) for Patients with Psychosis: A Pragmatic Cluster-Randomised Controlled Trial in Community Care. Psychother Psychosom..

[12] Tricco AC, Lillie E, Zarin W, O’Brien KK, Colquhoun H, Levac D (2018). PRISMA extension for scoping reviews (PRISMA-ScR): Checklist and explanation. Ann Intern Med.

[13] Peters MDJ, Godfrey CM, McInerney P, Baldini Soares C, Khalil H, Parker D (2015). Methodology for JBI Scoping Reviews. The Joanna Briggs Institute Reviewers’ Manual 2015.

[14] The EndNote Team (2014). EndNote X8.2.

[15] The Effective Public Health Practice Project. Quality Assessment Tool for quantitative studies. 1999. Available from: https://www.ephpp.ca/PDF/Quality%20Assessment%20Tool_2010_2.pdf. [Cited 2022 May 19].

[16] Thomas BH, Ciliska D, Dobbins M, Micucci S (2004). A process for systematically reviewing the literature: Providing the research evidence for public health nursing interventions. Worldviews Evid Based Nurs.

[17] Deeks JJ, Dinnes J, D’Amico R, Sowden AJ, Sakarovitch C, Song F, et al. Evaluating non-randomised intervention studies. Health Technol Assess (Rockv). 2003;7(27). Available from: www.hta.ac.uk/htacd.htm. [Cited 2022 May 19]. 10.3310/hta7270 14499048

[18] Critical Appraisal Skills Programme. CASP Qualitative Checklist. 2018. Available from: https://casp-uk.net/images/checklist/documents/CASP-Qualitative-Studies-Checklist/CASP-Qualitative-Checklist-2018_fillable_form.pdf. [Cited 2022 May 19].

[19] Long HA, French DP, Brooks JM (2020). Optimising the value of the critical appraisal skills programme (CASP) tool for quality appraisal in qualitative evidence synthesis. Res Methods Med Health Sci.

[20] Moreno-Peral P, Conejo-Cerón S, de Dios LJ, King M, Nazareth I, Martín-Pérez C (2021). Use of a personalised depression intervention in primary care to prevent anxiety: a secondary study of a cluster randomised trial. Br J Gen Pract.

[21] Bellón JÁ, Conejo-Cerón S, Moreno-Peral P, King M, Nazareth I, Martín-Pérez C (2016). Intervention to prevent major depression in primary care: a cluster randomized trial. Ann Intern Med.

[22] Fernández A, Mendive JM, Conejo-Cerón S, Moreno-Peral P, King M, Nazareth I (2018). A personalized intervention to prevent depression in primary care: Cost-effectiveness study nested into a clustered randomized trial. BMC Med..

[23] Mathieson F, Stanley J, Collings C, Tester R, Dowell A (2019). Cluster randomised controlled trial of a guided self-help mental health intervention in primary care. BMJ Open.

[24] Montag Schafer K, Gionfriddo MR, Boehm DH (2016). Shared decision making and medication therapy management with the use of an interactive template. J Am Pharm Assoc.

[25] Bellón JÁ, de Dios Luna J, King M, Moreno-Küstner B, Nazareth I, Montón-Franco C (2011). Predicting the onset of major depression in primary care: international validation of a risk prediction algorithm from Spain. Psychol Med..

[26] Lam CLK, Fong DYT, Chin WY, Lee PWH, Lam ETP, Lo YYC (2010). Brief problem-solving treatment in primary care (PST-PC) was not more effective than placebo for elderly patients screened positive of psychological problems. Int J Geriatr Psychiatry.

[27] Mathieson F, Mihaere K, Collings S, Dowell A, Stanley J (2012). Maori cultural adaptation of a brief mental health intervention in primary care. J Prim Health Care.

[28] Gask L, Dowrick C, Salmon P, Peters S, Morriss R (2011). Reattribution reconsidered: Narrative review and reflections on an educational intervention for medically unexplained symptoms in primary care settings. J Psychosom Res.

[29] Brody DS, Lerman CE, Wolfson HG, Craig Caputo G, Brody of Medicine. Improvement in physicians’ counseling of patients with mental health problems. Arch Intern Med. 1990;150:993–8. Available from: http://archinte.jamanetwork.com/. 2331204

[30] Collings S, Mathieson F, Dowell A, Stanley J, Jenkin G, Goodyear-smith F (2012). Acceptability of a guided self-help mental health intervention in general practice. Fam Pract.

[31] Gask L, Goldberg D, Porter R, Creed F. The treatment of somatization: Evaluation of a teaching package with general practice trainees. J Psychosom Res. 1989;33(6):697–703. 10.1016/0022-3999(89)90085-8. 10.1016/0022-3999(89)90085-8 2621673

[32] Jerant A, Kravitz RL, Azari R, White L, García JA, Vierra H (2009). Training residents to employ self-efficacy-enhancing interviewing techniques: Randomized controlled trial of a standardized patient intervention. J Gen Intern Med.

[33] Jerant A, Kravitz RL, Tancredi D, Paterniti DA, White L, Baker-Nauman L (2016). Training primary care physicians to employ self-efficacy-enhancing interviewing techniques: randomized controlled trial of a standardized patient intervention. J Gen Intern Med.

[34] Jerant A, Lichte M, Kravitz RL, Tancredi DJ, Magnan EM, Hudnut A (2016). Physician training in self-efficacy enhancing interviewing techniques (SEE IT): Effects on patient psychological health behavior change mediators. Patient Educ Couns.

[35] Mathieson F, Collings S, Dowell A, Goodyear-Smith F, Stanley J, Hatcher S. Collaborative research: A case example of dissemination of CBT in primary care. Cogn Behav Therap. 2013;6:E4. 10.1017/S1754470X13000093.

[36] Morriss R, Gasak L, Ronalds C, Downes-Grainger E, Thompson H, Leese B (1998). Cost-effectiveness of a new treatment for somatized mental disorder taught to GPs. Fam Pract..

[37] Seal KH, Pyne JM, Manuel JK, Li Y, Koenig CJ, Zamora KA (2021). Telephone veteran peer coaching for mental health treatment engagement among rural veterans: the importance of secondary outcomes and qualitative data in a randomized controlled trial. J Rural Health.

[38] Mathieson F, Stanley J, Collings C (Sunny), Tester R, Dowell A. Cluster randomised controlled trial of a guided self-help mental health intervention in primary care. BMJ Open. 2019;9(2):e023481. Available from: https://bmjopen.bmj.com/content/9/2/e023481. [Cited 2021 Sep 29]. 10.1136/bmjopen-2018-023481 PMC6398763 30819700

[39] Griffin SJ, Kinmonth AL, Veltman MWM, Gillard S, Grant J, Stewart M (2004). Effect on Health-Related Outcomes of Interventions to Alter the Interaction Between Patients and Practitioners: A Systematic Review of Trials. Ann Fam Med..

[40] Menichetti J, Lie H, Mellblom AV, Brembo EA, Lie HC, Mellblom A v, et al. Tested communication strategies for providing information to patients in medical consultations: a scoping review and quality assessment of the literature. Elsevier. 2021. Available from: https://www.sciencedirect.com/science/article/pii/S073839912100046X. [Cited 2022 Mar 24]. 10.1016/j.pec.2021.01.019 33516591

[41] Hutton C, Gunn J. Do longer consultations improve the management of psychological problems in general practice? A systematic literature review. BMC Health Serv Res. 2007;7. Available from: https://www.embase.com/search/results?subaction=viewrecord&id=L46896053&from=export. 10.1186/1472-6963-7-71 PMC1890290 17506904

[42] Stanton E. The case for change for British mental healthcare. Available from: http://www.hsj.co. [Cited 2023 Mar 11]. 10.1177/0141076814522144 PMC4109332 24692410

[43] Levinson W, Gorawara-Bhat R, Lamb J (2000). A study of patient clues and physician responses in primary care and surgical settings. J Am Med Assoc.

[44] Kyanko KA, Curry LA, Keene DE, Sutherland R, Naik K, Busch SH (2021). Does Primary Care Fill the Gap in Access to Specialty Mental Health Care? A Mixed Methods Study.

[45] Parker D, Byng R, Dickens C, McCabe R. "Every structure we’re taught goes out the window": General practitioners’ experiences of providing help for patients with emotional concerns'. Health Soc Care Community. 2020;28(1):260–9. 10.1111/hsc.12860. 10.1111/hsc.12860 PMC6916159 31621140

[46] Saini P, Chantler K, Kapur N (2016). General practitioners’ perspectives on primary care consultations for suicidal patients. Health Soc Care Community.

[47] Hyde J, Calnan M, Prior L, Lewis G, Kessler D, Sharp D (2005). A qualitative study exploring how GPs decide to prescribe antidepressants. Br J Gen Pract.

[48] Tyrell A. A Review of Innovative and Extended Roles within Mental Health Pharmacy. NHS Health Education England. 2020. Available from: https://www.hee.nhs.uk/sites/default/files/documents/Pharmacy%20Extended%20Roles%20Report.pdf.

[49] Garzonis K, Mann E, Wyrzykowska A, Kanellakis P (2015). Improving patient outcomes: effectively training healthcare staff in psychological practice skills: a mixed systematic literature review. Eur J Psychol.

[50] Choy-Brown M, Stanhope V (2018). The availability of supervision in routine mental health care HHS Public Access. Clin Soc Work J.

[51] Foster JM, Sawyer SM, Smith L, Reddel HK, Usherwood T (2015). Barriers and facilitators to patient recruitment to a cluster randomized controlled trial in primary care: Lessons for future trials. BMC Med Res Methodol..

[52] Moffat KR, Shi W, Cannon P, Sullivan F (2023). Factors associated with recruitment to randomised controlled trials in general practice: a systematic mixed studies review. Trials..

[53] Peters MDJ, Marnie C, Tricco AC, Pollock D, Munn Z, Alexander L (2021). Updated methodological guidance for the conduct of scoping reviews. JBI Evid Implement.

