# Supplementary material for: Structured communication methods for mental health consultations in primary care: a scoping review
Source: BMC Prim Care. 2023 Sep 4;24:175. doi: 10.1186/s12875-023-02129-y (PMC10476363; doi:10.1186/s12875-023-02129-y)
Supplement: Supplementary file 1 — Additional file 1. Keywords and index terms by database. [file 12875_2023_2129_MOESM1_ESM.docx]

**Additional file 1 - Keywords and index terms by database**

| **CINAHL** |
| --- |
| (structure* OR effectiv* OR framework OR ordered OR protocol OR systematic OR planned) AND (interaction OR communication OR conversation OR consultation OR meeting OR "decision aid" OR "action planning" OR "visit planning" OR "agenda setting" OR appointment) AND ("mental illness" OR "mental disorder" OR "mental condition" OR "mental disease" OR "mental ill health" OR "psychiatric illness" OR "psychiatric disorder" OR "psychiatric condition" OR "psychiatric disease" OR "psychiatric ill health" OR "psychological illness" OR "psychological disorder" OR "psychological condition" OR "psychological disease" OR "psychological ill health") AND (gp OR "general practitioner" OR "primary care" OR "family practice" OR "family doctor" OR "primary health care") |
| **Cochrane** |
| (structure* OR effectiv* OR framework OR ordered OR protocol OR systematic OR planned) AND (interaction OR communication OR conversation OR consultation OR meeting OR "decision aid" OR "action planning" OR "visit planning" OR "agenda setting" OR appointment) AND ("mental illness" OR "mental disorder" OR "mental condition" OR "mental disease" OR "mental ill health" OR "psychiatric illness" OR "psychiatric disorder" OR "psychiatric condition" OR "psychiatric disease" OR "psychiatric ill health" OR "psychological illness" OR "psychological disorder" OR "psychological condition" OR "psychological disease" OR "psychological ill health") AND (gp OR "general practitioner" OR "primary care" OR "family practice" OR "family doctor" OR "primary health care") |
| **EMBASE** |
| (structure* OR effectiv* OR framework OR ordered OR protocol OR systematic OR planned) AND (interaction OR communication OR conversation OR consultation OR meeting OR 'decision aid' OR 'action planning' OR 'visit planning' OR 'agenda setting' OR appointment) AND ('mental illness' OR 'mental disorder' OR 'mental condition' OR 'mental disease' OR 'mental ill health' OR 'psychiatric illness' OR 'psychiatric disorder' OR 'psychiatric condition' OR 'psychiatric disease' OR 'psychiatric ill health' OR 'psychological illness' OR 'psychological disorder' OR 'psychological condition' OR 'psychological disease' OR 'psychological ill health') AND (gp OR 'general practitioner' OR 'primary care' OR 'family practice' OR 'family doctor' OR 'primary health care') |
| **PsycInfo** |
| (structure* OR effectiv* OR framework OR ordered OR protocol OR systematic OR planned) AND (interaction OR communication OR conversation OR consultation OR meeting OR 'decision aid' OR 'action planning' OR 'visit planning' OR 'agenda setting' OR appointment) AND ('mental illness' OR 'mental disorder' OR 'mental condition' OR 'mental disease' OR 'mental ill health' OR 'psychiatric illness' OR 'psychiatric disorder' OR 'psychiatric condition' OR 'psychiatric disease' OR 'psychiatric ill health' OR 'psychological illness' OR 'psychological disorder' OR 'psychological condition' OR 'psychological disease' OR 'psychological ill health') AND (gp OR 'general practitioner' OR 'primary care' OR 'family practice' OR 'family doctor' OR 'primary health care') |
| **PUBMED** |
| Search ((((((((structur* OR effect* OR order*))) AND (((((interact*) OR communicat*) OR conversat*) OR meet*)))) OR (("decision aid" OR "action plan*" OR "visit plan*" OR "agenda set*")))) AND ((((((((((Primary Health Care[MeSH Terms]) OR Physicians, Primary Care[MeSH Terms] OR Physicians, Family[MeSH Terms]))) OR GP[Title/Abstract]) OR "general practitioner*"[Title/Abstract]) OR "family physician*"[Title/Abstract]) OR "family practic*"[Title/Abstract]) OR "family doctor*"[Title/Abstract])) OR "primary healthcare physician"[Title/Abstract])) AND (((((((mental[Title/Abstract]) OR psychiatr*[Title/Abstract]) OR long-term[Title/Abstract]) OR longterm[Title/Abstract]) OR chronic[Title/Abstract])) AND (((((illness*[Title/Abstract]) OR disorder*[Title/Abstract]) OR condition*[Title/Abstract]) OR diseas*[Title/Abstract]) OR "ill health"[Title/Abstract])) |

CINAHL - Cumulative Index to Nursing and Allied Health Literature; EMBASE - Excerpta Medica Database
