# Supplementary material for: Structured communication methods for mental health consultations in primary care: a scoping review
Source: BMC Prim Care. 2023 Sep 4;24:175. doi: 10.1186/s12875-023-02129-y (PMC10476363; doi:10.1186/s12875-023-02129-y)
Supplement: Supplementary file 2 — Additional file 2. Quality assessment by study. [file 12875_2023_2129_MOESM2_ESM.docx]

**Additional file 2 – Quality assessment by study**

| **Study** | **Criteria** | | | | | | | | | | | | | |  |
| --- | --- | --- | --- | --- | --- | --- | --- | --- | --- | --- | --- | --- | --- | --- | --- |
| *Quantitative studies*  *(EPHPP tool)* | **A** | **B** | **C** | **D** | **E** | **F** | **G1** | **G2** | **G3** | **H1** | **H2** | **H3** | **H4** | **GLOBAL RATING** | |
| **Bellon et al 2016; Fernandez et al 2018** | Moderate | Strong | Moderate | Strong | Strong | Strong | 80-100 | No | No | practice/office | individual | Yes | Yes | Strong | |
| **Brody et al 1990** | Strong | Strong | Strong | Moderate | Weak | Weak | Can't tell | No | Can't tell | practice/office | individual | Yes | Can't tell | Weak | |
| **Collings et al 2012** | Moderate | Moderate | N/A | Moderate | Weak | Strong | 80-100 | No | No | individual | individual | Yes | Yes | Moderate | |
| **Gask et al 1989** | Moderate | Moderate | N/A | Moderate | Moderate | Strong | 80-100 | No | No | Organisation/institution | Individual | Yes | No | Moderate | |
| **Jerant et al 2009** | Weak | Strong | Strong | Moderate | Weak | Strong | 80-100 | Yes | No | individual | individual | Yes | Can't tell | Weak | |
| **Jerant et al 2016a** | Weak | Strong | Strong | Moderate | Weak | Strong | 80-100 | Yes | No | individual | individual | Yes | No | Weak | |
| **Jerant et al 2016b** | Weak | Moderate | Moderate | Moderate | Moderate | Strong | 80-100 | No | Can’t tell | Individual | Individual | Yes | No | Moderate | |
| **Lam et al 2009** | Moderate | Strong | Strong | Moderate | Strong | Moderate | 60-79 | Yes | Yes | individual | individual | Yes | Yes | Moderate | |
| **Mathieson et al 2019** | Moderate | Strong | Strong | Moderate | Strong | Strong | 80-100 | No | Yes | practice/office | individual | Yes | Yes | Moderate | |
| **Morriss et al 1998** | Moderate | Weak | Strong | Moderate | Strong | Strong | 80-100 | No | Can’t tell | Practice/office | Individual | Yes | No | Moderate | |
| *Mixed studies*  *(EPHPP tool)* |  |  |  |  |  |  |  |  |  |  |  |  |  |  | |
| **Mathieson et al 2012** | Moderate | Moderate | N/A | Weak | Moderate | Weak | less than 60 | No | No | individual | individual | Yes | Yes | Weak | |
| **Schafer et al 2016** | Moderate | Moderate | N/A | Weak | Weak | Strong | 80-100 | No | No | individual | individual | Yes | Yes | Weak | |
| **Seal et al 2021** | Weak | Strong | Strong | Moderate | Strong | Moderate | 60-79 | Yes | Can't tell | individual | individual | Yes | No | Moderate | |
|  | | | | | | | | | | | | | | |  |
| *Qualitative study*  *(CASP checklist)* | **1 Clear aims** | **2 Appropriate methodology** | **3 Appropriate design** | **4 Appropriate recruitment** | **5 Data collection** | **6 Relationship** | **7 Ethical considerations** | **8 Data analysis** | **9 Findings** | **10 Value** |  |  |  |  | |
| **Mathieson et al 2013** | Yes | Yes | Yes | Yes | Yes | Yes | No | No | Yes | Example of how to use collaborative research practice to improve uptake in real world practice | | | | | |

EPHPP - Effective Public Health Practice Project; CASP - Critical Appraisal Skills Programme
